# Suicide by poisoning in Pakistan: review of regional trends, toxicity and management of commonly used agents in the past three decades

**DOI:** 10.1192/bjo.2021.923

**Published:** 2021-06-17

**Authors:** Maria Safdar, Khalid Imran Afzal, Zoe Smith, Filza Ali, Pervaiz Zarif, Zahid Farooq Baig

**Affiliations:** Department of Forensic Medicine, Postgraduate Medical Institute, Pakistan; Department of Psychiatry and Behavioral Neuroscience, University of Chicago, Illinois, USA; Department of Psychology, Loyola University, Illinois, USA; Department of Forensic Medicine, CMH Multan Institute of Medical Sciences, Pakistan; Department of Forensic Medicine, Postgraduate Medical Institute, Pakistan; Department of Medicine, CMH Lahore Medical College and Institute of Dentistry, Pakistan

**Keywords:** Low- and middle-income countries, suicide, mortality, epidemiology, self-harm

## Abstract

**Background:**

Suicide is one of the leading mental health crises and takes one life every 40 seconds. Four out of every five suicides occur in low- and middle-income countries. Despite religion being a protective factor against suicide, the estimated number of suicides is rapidly increasing in Pakistan.

**Aims:**

Our review focuses on the trends of suicide and means of self-poisoning in the past three decades, and the management of commonly used poisons.

**Method:**

We searched two electronic databases (PubMed and PakMediNet) for published English-language studies describing agents used for suicide in different regions of Pakistan. A total of 46 out of 85 papers (*N* = 54 747 cases) met our inclusion criteria.

**Results:**

Suicidal behaviour was more common among individuals younger than 30 years. Females comprised 60% of those who attempted suicide in our study sample, although the ratio of completed suicides favoured males. There were regional trends in the choice of agent for overdose. Organophosphate poisoning was reported across the nation, with a predominance of cases from the agricultural belt of South Punjab and interior Sindh. Aluminium phosphide (‘wheat pills’) was a preferred agent in North Punjab, whereas paraphenylenediamine (‘*kala pathar*’) was implicated in deaths by suicide from South Punjab. Urban areas had other means for suicide, including household chemicals, benzodiazepines, kerosene oil and rat poison.

**Conclusions:**

Urgent steps are needed, including psychoeducational campaigns on mental health and suicide, staff training, medical resources for prompt treatment of self-poisoning and updated governmental policy to regulate pesticide sales.

Suicide is the second leading cause of death in 15- to 29-year-olds globally, and 10- to 34-year-olds in the USA.^1,2^ The World Health Organization (WHO) estimates that 800 000 people die by suicide every year, which translates into one death every 40 seconds, and 79% of global suicides occur in low- and middle-income countries (LMICs).^3^ The World Bank Atlas defines low-income countries as having a gross national income (GNI) per capita of $1025 or less in 2018, and lower-middle-income countries as having a GNI per capita of $1026–$3995.^4^ Although pesticide ingestion, hanging and firearms are among the most common methods of suicide worldwide,^1^ trends vary between nations regarding the age groups, access and availability of the means.^5–8^ Mirroring global studies, the three most common methods for suicides in Pakistan are poisoning, firearms and hanging.^9,10^

## Pakistan

Pakistan is the fifth most populous country in the world.^11^ It is predominantly an agricultural country and, according to the 2017 National Census, around 64% of its population of 207 million is considered rural.^12,13^ The population ratio favours males (51.23%), with a male:female ratio of 1.05.^12^ About 50% of the population is under 20 years of age, and 35% is under 15 years of age. The literacy rate of Pakistan, as measured by the ability of people aged ≥15 years to read and write, is around 59%, which is lower than the average literacy rate in other South Asian countries (71.70%) and for LMICs overall (75%).^14^ Men have a literacy rate of 71%, whereas women have a literacy rate of <47%.^12,14,15^ The literacy rate in large urban centres such as Karachi and Lahore, the two largest cities in the country, is close to 75%, whereas the average literacy rate in rural areas is <50%.^16^ Along with other factors, terrorism has negatively affected sustained economic growth in Pakistan over the past two decades, leading to a high unemployment rate. The health indicators of the country continue to remain poor.^17–20^

## Geography and demography

**Fig. 1:**
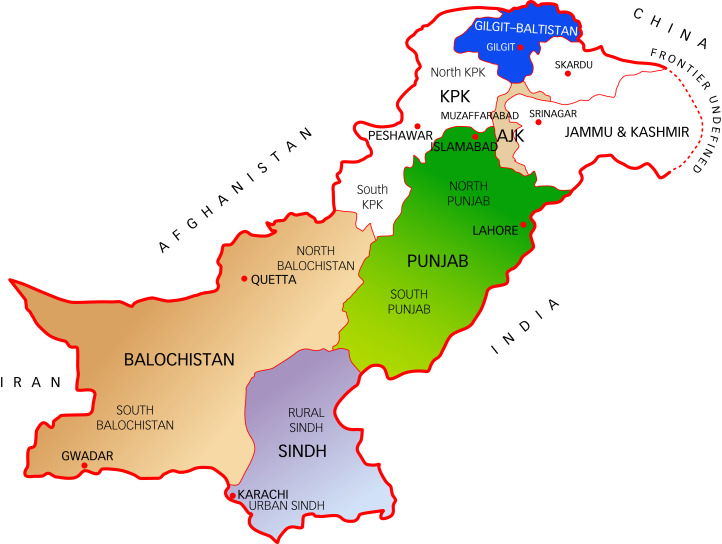
Geographical map of Pakistan. AJK, Azad Jammu and Kashmir; KPK, Khyber Pakhtunkhwa.

Geographically, the country is composed of four provinces – Punjab, Sindh, Balochistan, and Khyber Pakhtunkhwa (KPK) – and Gilgit–Baltistan, a newly created province in the north (Fig. 1).^21,22^ The Punjab and Sindh are fertile plains with agriculture-based economies. Balochistan and KPK are bound by strong tribal traditions. Gun ownership is a shared pride between the two provinces. Balochistan is rugged, rich in minerals and mostly barren.^23,24^ In the north of Pakistan, Gilgit–Baltistan is home to three large mountainous ranges: the Himalayas, the Karakoram and the Hindu Kush. The scenic region has beautiful valleys and river-irrigated lands.^21,25^ Shah and Amjad^26^ measured the cultural diversity of different regions of Pakistan. They found a high masculinity index score in all provinces, indicating a difference in social genders, with clear-cut roles. Uncertainty avoidance index scores were low in all provinces, mainly because a firm belief in Allah (God Almighty) led to most people not feeling threats or uncertainty about the future. Individualism index scores were low in all provinces, especially in KPK and Balochistan, signifying collectivism as a national culture. The people of Pakistan possessed a strong urge toward group cohesiveness and the expectation of loyalty.^26^

Approximately 96% of the population of Pakistan is Muslim.^27,28^ Like other major religions, Islam condemns suicide, declaring it an unforgivable sin.^29–31^ This could be a significant deterrent to suicide, evidenced by the traditionally low rates reported in Muslim countries compared with non-Muslim countries.^32^ Based on religious tenets, both suicide and self-harm are illegal and punishable by imprisonment and fines under Pakistani law, adding another deterrent to suicide.^33–35^ Studies from other LMICs and higher-income (GNI per capita of ≥$12 376) Muslim-majority countries also show a lower suicide rate than non-Muslim-majority countries.^36,37^ Arya et al describe the geographical heterogeneity of suicide rates in the neighbouring LMIC of India, focusing on religion, caste, tribe, etc. The authors found that the rate of suicide was lowest for Sikhs and Muslims, and highest for Hindus and Christians.^38^

## Suicide statistics

Pakistan has no vital registrations and lacks accurate figures for death by suicide.^39^ As compared with the 2017 global suicide death rate per 100 000 people for both genders of 9.98,^40^ the estimated age-standardised suicide rate in Pakistan is 4.4 per 100 000 people.^41^ The suicide death rates in neighbouring India, Bangladesh and Sri Lanka are 13.33, 5.73 and 7.55 per 100 000 people, respectively. Despite the low estimated rate, recent data suggest that suicide is becoming a significant public health problem in Pakistan.^42–45^ The WHO published a report showing an increase in the reported suicide rate of 2.6% from the year 2000.^1^ Because of the social, legal and religious factors noted above, suicide and self-harm are not reported or are underreported. Recent reports have shown rapidly increasing rates for suicide and self-harm across the country.^34,42,46^ Shekhani et al noted a stigmatisation of suicidal behaviour contributing toward a lack of research on the subject.^10^ We did not find literature on suicide or self-harm that compared different regions of Pakistan or differentiated between urban and rural populations.

To address the gap in current knowledge, this is the first study to map the regional trends of suicide by poisoning in Pakistan, and detail urban versus rural differences. We also aim to provide a detailed account of the pathophysiology and management strategies of agents used in suicide attempts, to give readers a comprehensive review on the subject. Our analysis will provide future research directions and inform policy for suicide prevention in Pakistan, focusing on regional and urban versus rural differences in suicide attempts.

## Method

We searched two electronic databases (PubMed and PakMediNet) for studies describing agents used for suicide in different regions of Pakistan, using the following terms: suicide, death, poisoning, drugs, overdose and Pakistan. We considered studies published in the English language within the past 30 years, and conducted the search from October to December 2019. Our null hypothesis was that there is no regional or urban versus rural difference in suicide by poisoning in Pakistan. We included primary research, case series and case reports, focusing on different agents used by adults of both genders, aged ≥18 years, who attempted suicide. Studies involving ex-pat Pakistanis and those using means of suicide other than overdose were excluded. We did not include single case reports as most focused on uncommon means of death or unusual clinical presentations that were not the focus of our study. The Postgraduate Medical Institute at Lahore, Pakistan, approved all of the data collection for this research project according to its policies regarding studies involving human patients.

After retrieving 85 articles from both databases, two independent reviewers screened the titles and abstracts for relevance. Sixty-two papers met the inclusion criteria; however, sixteen were case reports and were not included. Most studies were descriptive, with only three that used a case–control design. The majority of the studies were from urban areas (74%) and addressed determinants rather than risk factors. The WHO defines determinants as a range of behavioural, biological and socioeconomic factors that influence the health of populations.^47^ The risk factors are characteristics or attributes within an individual that influence the likelihood of disease.^10^ Most studies reported gender (95.3%) and age (93.0%) differences. We identified eight distinctive regions, including North and South Punjab, North and South KPK, interior Sindh (all cities except Karachi), urban Sindh (represented by studies from the largest city of Karachi), Balochistan and Gilgit–Baltistan (Table 1). The four predominant agents used in the attempted and completed suicides were organophosphates, aluminium phosphide (or ‘wheat pills’), paraphenylenediamine (or ‘*kala pathar*’) and others (including over-the-counter medications and household chemicals). We describe the clinical presentation, pathophysiological mechanism, morbidity, mortality and available treatments in the Discussion section. The authors assert that all procedures contributing to this work comply with the ethical standards of the relevant national and institutional committees on human experimentation and with the Helsinki Declaration of 1975, as revised in 2008.

**Table 1:** Studies on commonly used agents for poisoning in Pakistan, by region

Region	City	Reference	Total cases	Age (mean + s.d./range/ median)^	Gender, male:female	Poison used	Mortality	Study type
Organophosphate (25 articles published)								
North Punjab (5 articles)	Wah Cantt^a^	Bhatti N, et al.^48^	126	25.9 ± 9.75	48:52	Drugs 58%, organophosphates 18%, wheat pills 10%, corrosives 6%	–	Descriptive
	Rawalpindi	Maqbool F, et al.^49^	62	23.3 ± 6.1	53:47	Organophosphates	–	Descriptive
	Rawalpindi^a^	Khurram M, et al.^50^	85	24.35 ± 7.69	41:59	Medicine 53%, organophosphates 21%, corrosives 10%	2.5%	Descriptive
	Mianwali^a^	Tahir MN, et al.^51^	108	11–40	78:22	Toxic substance 36%, pesticides 31%, drug overdose 11%	–	Descriptive
	Lahore^a^	Naheed T, et al.^52^	114	25.89 ± 11.48	53:47	Urban: household toxins and drugs; rural: wheat pills and organophosphates	–	Descriptive
North KPK (3 articles)	Peshawar^a^	Ali Z, et al.^53^	128	25.79 ± 11.23	40:60	Organophosphates 31%, benzodiazepine 13%, wheat pills 11%	Total 12–44%	Descriptive
	Peshawar^a^	Rahim F, et al.^54^	92	26.8 ± 13.9	40:60	Medicine 53%, organophosphates 36%, wheat pills 11%	25%	Descriptive
	Peshawar	Bilal M, et al.^55^	50	30.88 + 15.72	54:46	Organophosphates	10%	Descriptive
Interior Sindh (4 articles)	Hyderabad	Shaikh MA.^56^	100	37.5 ± 9.5 (43 median)	78:22	Organophosphates	18%	Descriptive
	Nawabshah	Imran S, et al.^57^	387	26.14 ± 10.09	63:37	Organophosphates	27%	Descriptive
	Nawabshah	Faiz MS, et al.^58^	300	32 ± 5.2	17:83	Organophosphates	17%	Descriptive
	Jamshoro	Shaikh MA, et al.^59^	200	38.4 ± 3.5 (45 median)	81:19	Organophosphates	20%	Descriptive
Urban Sindh (12 articles)	Karachi^a^	Amir A, et al.^60^	2546	26.57 ± 11.82	51:49	Organophosphates 46%, off-label products 18%, rat poison 11%	4%	Descriptive
	Karachi^a^	Khan NU, et al.^61^	705	4–32 (21 median)	–	Drugs 24%, pesticides 15%, household toxins 9%	--	Descriptive
	Karachi	Ahmed A, et al.^62^	248	27.28 ± 11.5	27:73	Organophosphates	14%	Descriptive
	Karachi^a^	Imtiaz F, et al.^63^	11 925	14–22 (20 median)	34:66	Organophosphates 62%, kerosine oil 14%, drugs 23%	18%	Descriptive
	Karachi^a^	Asghar SP, et al.^64^	40	12–56 (30 median)	20:80	Organophosphates	Nil	Descriptive
	Karachi^a^	Bashir F.^65^	374	25 ± 10.1	62:38	Organophosphates 47%, corrosives 14%, drugs 23%	--	Descriptive
	Karachi	Ali P, et al.^66^	100	28.6 + 9.8	68:32	Organophosphates	20%	Case Series
	Karachi	Ather N, et al.^67^	2708	–	51:49	Organophosphates	5%	Descriptive
	Karachi^a^	Turabi A, et al.^68^	3334	8–50 (30 median)	50:50	Organophosphates 60%, corrosives 13%, rodenticides 7%, kerosine oil 2%	–	Descriptive
	Karachi	Hussain AM, et al.^69^	52	–	–	Organophosphates	8%	Descriptive
	Karachi^a^	Jamil H.^70^	1900	11–30	47:53	Organophosphates 40%, tranquilisers 21%, sedatives/hypnotics 10%	5.6%	Descriptive
	Karachi	Jamil H.^71^	755	11–30	40:60	Organophosphates	–	Descriptive
Balochistan (1 article)	Quetta	Khan NK, et al.^72^	46	15–35	Nil:100	Organophosphates	–	Descriptive
Wheat pills (9 articles published)								
North Punjab (7 articles)	Rawalpindi	Hassan A, et al.^73^	77	16–60	47:53	Wheat pills	33%	Descriptive
	Kharian	Iftikhar R, et al.^74^	52	25.10 ± 5.35	48:52	Wheat pills	87%	Descriptive
	Lahore	Ghazi MA.^75^	--	25 ± 5	--	Wheat pills	70%	Review
	Lahore^a^	Naheed T, et al.^52^	114	25.89 ± 11.48	53:47	Urban: household toxins and drugs; rural: wheat pills and organophosphates	–	Descriptive
	Lahore^a^	Shoaib S, et al.^76^	107	11–60	55:45	Wheat pills 33%, bleach 26%, benzodiazepines 19%	26%	Descriptive

	Lahore^a^	Asif A, et al.^77^	1390	11–90	69:31	Poison unknown 83%, rat poison pills 4%, wheat pills 3%	–	Descriptive
	Sahiwal	Qureshi MA, et al.^78^	110	12–40	41:59	Wheat pills	44%	Descriptive
North KPK (2 articles)	Peshawar^a^	Ali Z, et al.^53^	128	25.79 ± 11.23	40:60	Organophosphates 31%, benzodiazepines 13%. wheat pills 11%	Total 12–44%	Descriptive
	Peshawar^a^	Rahim F, et al.^54^	92	26.8 ± 13.9	40:60	Medicine 53%, organophosphates 36%, wheat pills 11%	25%	Descriptive
*Kala pathar* (11 articles published)								
South Punjab (7 articles)	Sahiwal	Akram A, et al.^79^	88	>14 (92%)	22:78	*Kala pathar*	–	Descriptive
	Multan	Tanweer S, et al.^80^	122	23.21 ± 8.2	20:80	*Kala pathar*	28%	Descriptive
	Multan	Haider SH, et al.^81^	32	21.06 ± 3.25	34:66	*Kala pathar*	28%	Descriptive
	Bahawalpur	Khan MA, et al.^82^	1258	5–63 (21 median)	65:35	*Kala pathar*	24%	Descriptive
	Bahawalpur	Ishtiaq R, et al.^83^	109	22 ± 3.4	29:71	*Kala pathar*	39%	Descriptive
	Bahawalpur	Qasim AP, et al.^84^	109	11–60	11:89	*Kala pathar*	21%	Descriptive
	Rahim Yar Khan	Akbar K, et al.^85^	65	24.35 ± 9.8	28:72	Kala pathar	--	Descriptive
						–	-
South KPK (2 articles)	Dera Ismail Khan	Ansari RZ, et al.^86^	503	12–39	21:79	*Kala pathar*	80%	Descriptive
	Dera Ismail Khan	Khan H, et al.^87^	38	22.08 ± 6.42	5:95	*Kala pathar*	47%	Descriptive
Interior Sindh (2 articles)	Hyderabad	Kazi MA et al.^88^	24	15–35	29:71	*Kala pathar*	42%	Descriptive
	Nawabshah	Khuhro BA, et al.^89^	16	25.87 ± 5.59	13:87	*Kala pathar*	38%	Descriptive
Over-the-counter drugs and household chemicals (17 articles published)								
North Punjab (6 articles)	Wah Cantt^a^	Bhatti N, et al.^48^	126	25.9 ± 9.75	48:52	Drugs 58%, organophosphates 18%, wheat pills 10%, corrosives 6%	–	Descriptive
	Rawalpindi^a^	Khurram M, et al.^50^	85	24.35 ± 7.69	41:59	Medicine 53%, organophosphates 21%, corrosives 10%	2.5%	Descriptive
	Mianwali^a^	Tahir MN, et al.^51^	108	11–40	78:22	Toxic substances 36%, pesticides 31%, drug overdose 11%	–	Descriptive
	Lahore^a^	Naheed T, et al.^52^	114	25.89 ± 11.48	53:47	Urban: household toxins and drugs; rural: wheat pills and organophosphates	–	Descriptive
	Lahore^a^	Shoaib S, et al.^76^	107	11–60	55:45	Wheat pills 33%, bleach 26%, benzodiazepines 19%	26%	Descriptive
	Lahore^a^	Asif A, et al.^77^	1390	11–90	69:31	Poison unknown 83%, rat poison pills 4%, wheat pills 3%	–	Descriptive
South Punjab (1 article)	Multan	Hashmi MU, et al.^90^	206	23.44 + 7.19	34:66	Corrosives	–	Descriptive
North KPK (2 articles)	Peshawar^a^	Ali Z, et al.^53^	128	25.79 ± 11.23	40:60	Organophosphates 31%, benzodiazepines 13%, wheat pills 11%	Total 12–44%	Descriptive
	Peshawar^a^	Rahim F, et al.^54^	92	26.8 ± 13.9	40:60	Medicine 53%, organophosphates 36%, wheat pills 11%	25%	Descriptive
Urban Sindh (8 articles)	Karachi^a^	Amir A, et al.^60^	2546	4–32 (21 median)	51:49	Organophosphates 46%, off-label products 18%, rat poison 11%	4%	Descriptive
	Karachi^a^	Khan NU, et al.^61^	705	14–22	–	Drugs 24%, pesticides 15%, household toxins 9%	--	Descriptive
	Karachi^a^	Imtiaz F, et al.^63^	11 925	25 + 10.1	34:66	Organophosphates 62%, kerosine oil 14%, drugs 23%	18%	Descriptive
	Karachi^a^	Bashir F, et al.^65^	374	25 + 10.1	62:38	Organophosphates 47%, corrosives 14%, drugs 23%	--	Descriptive
	Karachi^a^	Patel MJ, et al.^91^	324	36.2 ± 17.0	33:67	Benzodiazepines 55%, NSAIDs/analgesics 11%, antidepressants 10%	Nil	Descriptive
	Karachi^a^	Turabi A, et al.^68^	3334	8–50	50:50	Organophosphates 60%, corrosives 13%, rodenticides 7%, kerosine oil 2%	–	Descriptive
	Karachi^a^	Khan MM, et al.^92^	447	27.50 + 10.62	40:60	Drugs 73% (benzodiazepines 91%)	Nil	Descriptive
	Karachi^a^	Jamil H.^70^	1900	11–30	47:53	Organophosphates 40%, tranquilisers 21%, sedatives/hypnotics 10%	5.6%	Descriptive
Miscellaneous (2 articles published)								
North KPK (1 article)	Chitral	Ahmed Z, et al.^93^	168	10–50	38:62	Drowning 52%, hanging 26%, gunshot 17%, intoxication 5%	--	Descriptive
Gilgit–Baltistan (1 article)	Ghizer	Khan MM, et al.^94^	49	16–>70 Majority 16–35	Nil:100	Jumping into a river/lake 40%, intoxication 30%, strangulation 11%, firearm 5%	–	Descriptive

KPK, Khyber Pakhtunkhwa; NSAID, non-steroidal anti-inflammatory drug.

a. Studies cited more than once because of multiple toxins/drugs involved.

## Results

Table 1 shows the distribution of studies according to the regions, with details on study design, cohort size, gender, mean age, geographical region, city, suicide attempts or completion methods, and mortality. The exact doses of agents used in the suicide attempts were inconsistently reported and were not statistically meaningful for our study. The majority of the studies were from urban areas (74%) and addressed determinants rather than risk factors. Most studies reported the gender (95.3%) and age (93.0%) of the individuals. We identified eight distinctive regions, including North and South Punjab, North and South KPK, interior Sindh (all cities except Karachi) and urban Sindh (represented by studies from the largest city Karachi), Balochistan and Gilgit–Baltistan (Table 1). The four predominant agents used in the attempted and completed suicides were organophosphates, aluminium phosphide, paraphenylenediamine and others (including over-the-counter medications and household chemicals). Of the 47 studies, 53.2% examined organophosphates (*n* = 25), 36.2% examined over-the-counter agents and household chemicals (*n* = 17), 23.4% examined *kala pathar* (*n* = 11), 19.1% examined wheat pills (*n* = 9) and 4.3% examined ‘intoxication’ without indicating the agent used (*n* = 2). Note that some studies examined multiple agents, so the total exceeds the number of studies included. With the exception of two studies, all papers were published in the past two decades.

The total number of cases across the 53 studies was 54 747 (see Table 2). A total of 60% of overall study participants were female and 40% were male. Suicidal behaviour was more common among individuals aged <30 years, with a mean age of 27.9 years. See Table 3 for more comprehensive demographic information. Urban Sindh had the most publications (14 studies; *n* = 25 458), followed by North Punjab (12 studies; *n* = 2319), South Punjab (7 studies; *n* = 1901), interior Sindh (6 studies; *n* = 1027), North KPK (4 studies; *n* = 438), South KPK (2 studies; *n* = 541) and one study each from Balochistan (*n* = 46) and Gilgit–Baltistan (*n* = 46) (see Table 4 for demographic information by region). The overall mortality rate, regardless of the method, was 24.5%. Organophosphates were the most widely reported agent (25 studies; *n* = 35 479), with an average mortality rate of 13.9% (11 studies; *n* = 2364). The highest average mortality rate was for wheat pills, at 44.7% (9 studies; *n* = 2070). The lowest average mortality rate was for over-the-counter agents and household chemicals (17 studies; *n* = 20 911), at 12.1%. For *kala pathar*, the average mortality rate was 38.6% (11 studies; *n* = 2364). See Table 5 for more demographic information by different agents.

**Table 2:** Overall demographic information for included studies

Demographics	Mean/percentage
Participants	*N* = 54 747
Studies	*N* = 53
Female:male	60%:40%
Age	27.9 years
Urban:rural area	74%:26%
Mortality rate	24.5%
**Regions**	**Number of participants**
Balochistan	46
Gilgit–Baltistan	49
Interior Sindh	1027
North Khyber Pakhtunkhwa	438
North Punjab	2319
South Khyber Pakhtunkhwa	541
South Punjab	1901
Urban Sindh	25 458
**Regions**	**Number of studies**
Balochistan	1
Gilgit–Baltistan	1
Interior Sindh	6
North Khyber Pakhtunkhwa	4
North Punjab	12
South Khyber Pakhtunkhwa	2
South Punjab	7
Urban Sindh	14

**Table 3:** Demographic information of included studies by region

Region/demographics	Mean/percentage
**Balochistan**	
Participants	*n* = 46
Female:male	100%:0%
Age	20.5 years
Mortality rate	Not reported
**Gilgit–Baltistan**	
Participants	*n* = 49
Female:male	100%:0%
Age	25.5 years
Mortality rate	Not reported
**Interior Sindh**	
Participants	*n* = 1027
Female:male	53.1%:46.9%
Age	32.5 years
Mortality rate	27%
**North Khyber Pakhtunkhwa**	
Participants	*n* = 438
Female:male	57%:43%
Age	28.5 years
Mortality rate	21%
**North Punjab**	
Participants	*n* = 2319
Female:male	49.5%:50.5%
Age	26.6
Mortality rate	43.8%
**South Khyber Pakhtunkhwa**	
Participants	*n* = 541
Female:male	87%:13%
Age	23.8 years
Mortality rate	63.5%
**South Punjab**	
Participants	*n* = 1901
Female:male	68.4%:31.6%
Age	23.4 years
Mortality rate	28%
**Urban Sindh**	
Participants	*n* = 25 458
Female:male	55.1%:46.9%
Age	27.5 years
Mortality rate	7.46%

**Table 4:** Demographic information of included studies by agent

Agent/demographics	Mean/percentage
**Organophosphates**	
Participants	*n* = 35 479
Studies	*n* = 25
Urban:rural	16%:84%
Female:male	52.3%:47.7%
Age	28.9 years
Mortality rate	13.9%
**Over-the-counter agents and household chemicals**	
Participants	*n* = 20 911
Studies	*n* = 17
Urban:rural	6%:94%
Female:male	51.6%:48.4%
Age	26.0 years
Mortality rate	12.1%
***Kala pathar***	
Participants	*n* = 2364
Studies	*n* = 11
Urban:rural	36%:64%
Female:male	74.8%:25.2%
Age	24.5 years
Mortality rate	38.6%
**Wheat pills**	
Participants	*n* = 2070
Studies	*n* = 9
Urban:rural	11%:89%
Female:male	50.9%:49.1%
Age	27.7 years
Mortality rate	44.7%
**Miscellaneous**	
Participants	*n* = 217
Studies	*n* = 2
Urban:rural	0%:100%
Female:male	81%:19%
Age	27.8 years
Mortality rate	Not reported

**Table 5:** Overview of commonly used poisons in Pakistan

Poison	Symptoms	Diagnosis	Management	Lethal dose	Mortality	Regional prevalence	Cost
Organophosphates	Bronchorrhea, bronchoconstriction, excessive sweating, constricted pupils, abdominal cramps, involuntary defaecation and urination, tachycardia, QT prolongation, headaches, dizziness, drowsiness, confusion, anxiety, slurred speech, ataxia, psychosis, convulsions, coma, hypotension and respiratory depression	Clinical	Supportive care; decontaminate the patient and prevent further absorption via the gut, eyes, skin or lungs; administer atropine followed by enzyme reactivation by pralidoxime	Depends on many factors	10–27%	All over Pakistan, but more prevalent in North Punjab and Sindh	1200–1600 PKR per litre
Aluminium phosphide	Epigastric pain, vomiting, diarrhoea, dizziness and dyspnoea, multiorgan failure involving the heart, kidneys, lungs and liver	Silver nitrate test	Supportive care as no antidote is available; gastric lavage with potassium permanganate and mineral or coconut oil; renal replacement therapy in the early stage may be helpful	150–500 mg	33–87%	North Punjab	700–1000 PKR per 500 mg
Paraphenylenediamine	Angioneurotic oedema, rhabdomyolysis causing myoglobinuria, cola-coloured urine, oliguria and acute tubular necrosis leading to acute renal failure	Clinical	Supportive care as no antidote is available; early tracheostomy; intravenous fluids to prevent renal failure; renal replacement therapy in cases where ATN develops	7–10 g	21–47%	South Punjab and South KPK	500 PKR per 10 g
Over-the-counter agents and household chemicals	May present with CNS depression, CNS stimulation or a mixed picture; the heart rate, blood pressure, body temperature, respiratory rate, skin clamminess, pupillary reaction and neuromuscular abnormalities would provide clues to the correct diagnosis	Clinical	Supportive care; decontamination and gastric lavage with activated charcoal antidote (flumazenil for benzodiazepines) should be used if available; haemodialysis, haemofiltration and exchange transfusion could facilitate the removal of some agents	Depends on agent used	2.5–25%	Urban areas	Diazepam is one of the most commonly used benzodiazepines; it is 37 PKR for 30 10-mg tablets

ATN, acute tubular necrosis; KPK, Khyber Pakhtunkhwa; CNS, central nervous system.

Studies from Karachi (i.e. urban Sindh) included 25 458 individuals, of whom 55.1% were women aged 20–43 years (mean age 27.5 years). The average overall mortality rate for this region was 7.46 and ranged from 0 to 42%. Most studies from Karachi (73.3%) found organophosphates as the agent chosen for death by suicide, with an average mortality rate of 9.33 (range 0–20%). Other agents were also examined, including benzodiazepines, off-label agents, pesticides, corrosives, kerosene oil, rat poison, non-steroidal anti-inflammatory drugs (NSAIDs)/analgesics, and antidepressants. Two studies found that 55–91% of 771 people chose benzodiazepines as the agent of choice for attempting suicide. However, benzodiazepine overdose was associated with a 0% mortality rate in these studies. One study found that 18% of 2546 individuals chose off-label agents, whereas another study found that 15% of 705 individuals chose pesticides. Two studies of 3708 individuals found that 13.5% used corrosives. Kerosene oil was examined in two studies, with 2–14% of 15 259 individuals using it to commit suicide. Finally, rat poison (11% of 2546 individuals), NSAIDs/analgesics (11% of 324 individuals) and antidepressants (10% of 324 individuals) were all examined in one paper.

Interior Sindh included six studies from three cities: Hyderabad, Jamshoro and Nawabshah. The latter two cities are rural. There were 1027 individuals aged 16–43 years (mean age 32.5 years), of whom 53.1% were female. The most commonly studied agent was organophosphates (66.6% of studies, 987 individuals), whereas the other two studies examine *kala pathar* (40 individuals). Mortality rates for organophosphates ranged from 17 to 27% (mean 20.5%), whereas aluminium phosphide (two studies; *n* = 40) was higher at 38–42% (mean 40%). Overall mortality rates for this region averaged at 27%.

Within North Punjab, a total of 2319 cases were noted in 12 studies, with a male:female gender ratio of 50.5%:49.5% favouring males. The age range was 20–40 years (mean age 26.6 years) across six cities (Kharian, Lahore, Mianwali, Rawalpindi, Sahiwal and Wah Cantt). Of these cities, Mianwali and Sahiwal are considered rural, and the other four are urban. The overall mortality rates range from 2.5 to 87%, with a mean percentage of 43.8%. In North Punjab, almost half of individuals who ingested wheat pills died by suicide, indicating the high lethality of the agent. The overall mortality rate for wheat pills ranged from 33 to 87%, with an average of 52%. Other agents examined in the region included organophosphates (four studies), corrosives (two studies), benzodiazepines (one study), generic agents (one study), medicine (one study), ‘toxic substance’ (one study), pesticides (one study), household toxins (one study), bleach (one study), *kala pathar* (one study) and rat poison (one study). Mortality rates were not reported for these agents.

There were 1901 cases in 7 studies from three cities in South Punjab (Bahawalpur, Multan and Rahim Yar Khan). This region consisted of all urban cities, although the healthcare facilities’ catchment area extends into vast agricultural lands. Women comprised 68.4% of the samples, with an age range of 21–30 years (mean age 23.4 years). All seven studies examined paraphenylenediamine (*kala pathar*) poisoning, with a mortality rate of 28% (ranging from 21 to 39%). Only one study examined corrosives as the substance of choice for overdose, but this study did not report mortality.

North KPK included four studies with 438 cases from two cities: Peshawar (urban) and Chitral (rural). Women comprised 57% of the reported cases, with an age range of 26–31 years (mean 28.5 years). Mortality rates ranged from 10 to 44%, with an average overall mortality rate of 21%. No clear choice of agent for overdose emerged; however, similar to urban Sindh, organophosphates were included in three of the four studies, with a prevalence rate of 31–36%. Aluminium phosphide and benzodiazepines were the agents of choice 11% and 13% of the time, respectively. Interestingly, one study included methods outside of poisoning, finding that only 5% of individuals preferred an overdose by agents compared with other methods (drowning 52%, hanging 26%, firearms 17%).

For South KPK, there were two studies, both from Dera Ismail Khan, which is a rural area. There were 541 participants across the two studies, of whom 87% were female, and the average age was 23.8 years (range 12–39 years). Both studies only examined *kala pathar*, finding a high overall mortality rate of 63.5% (range 47–80%). One study included only 38 participants, 95% of whom were female, with a mortality rate of 47%. The second study confirmed the findings of the first paper, with a much higher number of reported cases (503 cases). The number of men in the second study rose to 21%, and the mortality rate rose to around 80%.

In Balochistan, there was only one study examining agents used by people attempting suicide. This sample included only 46 female participants in Quetta, an urban centre and the largest city in the province. This study only examined organophosphates but did not report mortality rates.

The Gilgit–Baltistan region included only one study, in the city of Ghizer (a rural town). This study included 49 individuals, all of whom were female. The means of suicide included jumping into a body of water (40%), ingesting a poisonous agent (30%), strangulation (11%) and the use of a firearm (5%). Mortality rates were not reported in this study.

In summary, organophosphate poisoning was reported from all four provinces. However, organophosphates played a more substantial role in the cases of suicide reported from North Punjab and interior Sindh, where it accounted for up to 60% of reported cases. Aluminium phosphide (wheat pill) poisoning was noted in agent overdoses reported mainly from North Punjab and North KPK, whereas paraphenylenediamine (*kala pathar*) was primarily used in suicide from South Punjab, with some reports from South KPK and interior Sindh. Compared with the rural population (where pesticides and paraphenylenediamine were most common), the urban population chose more varied agents for overdose, including household chemicals (bleach, corrosives), medicines (sedatives, tranquilisers, NSAIDs, antidepressants), rat poison pills and other toxic substances. Other means of suicide, such as hanging (asphyxiation), gunshot and drowning, were not the focus of our paper. Some studies in our analysis reported the reason for the suicide attempt. Five themes emerged, including financial problems, family conflicts, illicit spousal relationships, serious medical illness and failed romance. Studies did not report risk factors for suicide consistently enough to allow for a complete analysis of regional or urban versus rural differences in these risk factors.

## Discussion

To our knowledge, this is the first study to focus on the regional difference in suicide by poisoning in Pakistan. The results also suggest urban versus rural differences in the choice of poison. We discuss determinants of suicide behaviour and comprehensive management strategies for commonly used agents, to address existing gaps in suicide literature.

Our study found that pesticides (organophosphates and aluminium phosphide) are the most frequently used agents for suicide across Pakistan. As noted above, agriculture is the backbone of Pakistan's economy. The main crops include cotton, wheat, rice, maize and sugarcane, in addition to a large variety of regional fruits and vegetables.^13,95^ The need to meet the ever-increasing demand is one of the driving forces of the phenomenal rise in pesticide use in farming and agriculture. It does not spare even the remote areas of Pakistan.^96,97^ Pesticides are regulated in Pakistan by the Agriculture Pesticide Ordinance of 1971 (amended up to 1997) and Agriculture Pesticides Rules of 1973.^98^ Pakistan's Agriculture and Research Council detailed several elements regarding registration, production, procurement, transportation, distribution, sale, storage, usage and the safe disposition of empty containers.^98^ There are also institutional arrangements for pesticide monitoring and research.^99^ However, pesticides are readily available, and their unrestricted use continues to be widespread.^100^ A sobering study from the Khoj Foundation in 2009 reported that Pakistan used 14 times more pesticides for wheat and rice crops than India. Furthermore, the researchers found:

‘Pesticides are often stored in living rooms, among cookware and plates, and the bags in which they are sold are sometimes reused and sewn into quilts or floor covering. Utensils used to mix pesticides are often also used for cooking. They found that because women are not involved in the decision making around pesticide use and work both in the fields and in the home where pesticides are stored, they are at increased risk of poisoning.’^101^

Corresponding to these findings, several studies have investigated suboptimal or a complete lack of knowledge and awareness of pesticide hazards in these regions.^102–104^ Although unintentional poisoning is beyond the scope of this paper, this information is crucial in providing a glimpse of the problem and how it relates to easy accessibility and means for self-harm and suicide.

In our analysis, organophosphate overdose was reported in studies from across Pakistan, with the highest number of cases from the Punjab and Sindh regions (Table 5). Twelve studies were from Karachi, representing urban Sindh. We believe that, being the largest city of the province and Pakistan, Karachi receives patients with suicide overdose from all over Sindh, to receive care in its well-equipped medical institutions.^105^ Thus, the number of organophosphate poisonings from Karachi likely represents rural rather than urban Sindh. Similarly, studies from other metropolitan cities, such as Lahore or Rawalpindi in Punjab, treated patients with poisoning who were transferred from the surrounding rural areas to receive treatments. In the wheat-growing regions of North Punjab, aluminium phosphide or wheat pills are more readily available and were the most common agents to attempt suicide. North KPK also reported a high incidence of aluminium phosphide use.

Interestingly, there was no report of aluminium phosphide overdose from urban or interior Sindh, indicating that availability could be the critical factor in the choice of agent in suicide. As opposed to inhalational or skin contact in unintentional poisoning, ingestion was the most common method for suicide by pesticide.^106–109^ The chemical structure and management of organophosphates and aluminium phosphide poisoning are discussed later in the paper.

Paraphenylenediamine is an ingredient in a compound commonly known as *kala pathar* (Black Stone) in Urdu. It is used as a chemical ingredient in temporary tattoo ink, fabrics, dark makeup, photocopying inks, printing, rubber products and gasoline. In the Indian subcontinent and North Africa, paraphenylenediamine is an ingredient of black henna, which is used for hair dye and tattoo ink.^110–112^ Paraphenylenediamine was noted as the agent of choice for suicide in South Punjab, South KPK and interior Sindh. Its easy availability, unrestricted sale as a hair dye and the associated low cost of 10 PKR for a single dose (1 USD = 160.36 PKR (at the time of publication)) are the likely reasons behind the increasing number of cases in recent years.^113^ The ease of preparing the suicide concoction by mixing *kala pathar* in water increases the probability of its use in poisoning.^114^ Following the increasing number of cases, a unified social and print media campaign against the rapidly rising number of suicides with *kala pathar* led Punjab's government to issue a temporary ban on its open trading in September 2017 in South Punjab. In April 2018, the Punjab government expanded the temporary ban on *kala pathar* throughout the whole province.^114–116^ The management of paraphenylenediamine poisoning is discussed later.

We found significant differences in the choice of agents for suicide in urban versus rural populations (see Table 4). *Kala pathar* was used in 36% of overdose cases in the urban areas as opposed to 64% from the rural regions. More than 85% of the poisoning cases choosing organophosphates, aluminium phosphide and miscellaneous agents were from rural areas, whereas 94% of over-the-counter poisoning cases were from urban areas. Over-the-counter agents included drugs/medicines (benzodiazepines, tranquilisers, NSAIDs/analgesics, antidepressants, etc.), household toxins (bleach, rat poison pills or rodenticides, insecticides) and kerosene oil. The availability, accessibility and ease of use appeared to be significant factors influencing the choice of agents for suicide in our study.

Interestingly, the gender distribution was relatively similar for all agents except *kala pathar,* which favoured females (74% female *v.* 25% male). The category of ‘miscellaneous agents’ was mostly reported in males (19% female *v.* 81% male). Drowning or jumping into a lake or a river was a preferred method for suicide in North KPK and Gilgit–Baltistan, where there is ready access to rivers, lakes and streams. Except for North Punjab, where the female:male suicide ratio is almost equal, all other reported regions showed a higher incidence of reported suicide in females compared with males (see Table 3). The average age of suicide in our data was 27.9 years, with the youngest reported age of 20.5 years in Balochistan.

Suicide is a complex phenomenon, and its identity is often shrouded in mystery. Unspoken religious and cultural factors, especially in LMICs, may contribute toward its inadequate understanding, and Pakistan is no exception.^116^ Our study highlights social determinants such as financial problems, gender and cultural stressors influencing suicide. Although not reported in all of the studies, we identified economic issues, family conflicts, illicit spousal relationships, serious illness and failed romance as commonly identified reasons for suicide. Pakistan is an economically strained country with a high unemployment rate.^19^ Previous reports from the region similarly found a range of socially and culturally specific family problems, typically involving spouses, in-laws, parent–child conflicts, unfulfilled expectations at work or failure in school, and mental turmoil to be factors in suicide attempts.^116^ Pakistan's regional differences influence the execution of cultural norms. As discussed earlier, a low individualism index promotes collective culture, and a high masculinity index defines boundaries and gender roles.^26^ A deviation from tribal tradition could lead to a sense of betrayal among other clan members that can incite violence, especially against women.^10^ We found that all reported cases of suicide from Balochistan and Gilgit–Baltistan were females. In a recent news report, female suicides in the region were associated with the lack of freedom in choosing potential husbands.^117^

Ali et al suggest domestic and social issues as the most common reason for overdose accounting for up to 70% of the cases.^118^ In comparison, prior psychiatric history of suicide was possibly linked with suicide attempts in only 10% of the patients.^54^ As opposed to high-income countries, where primary psychiatric disorders such as major depression are often reported to be present in 80%–90% of deaths by suicide, in Pakistan, a premorbid mental health diagnosis is often absent.^119^ Treatment could potentially be delayed, as the patient's history, although very important, is often unreliable in suicide attempts.^120,121^ Fear of persecution, stigma and confidentiality around such a sensitive issue may lead to the concealment of facts, both by the patient and the family.^122^

Gender inequality and discrimination are significant issues both globally and in Pakistan.^123^ The country has a deep-rooted patriarchal culture with unequal gender role expectations.^124–126^ Women are expected to do household chores for the extended family. Men are the primary authority figures and considered the traditional breadwinners, which gives them a superior position to women. Although an increasing number of women are economically active, both in rural and urban areas, society has yet to recognise their contribution.^23^ Women are seldom included in decision-making and continue to be victims of abuse.^125,127^ Lack of gender-sensitive policies seems to hinder equitable political and economic status, birth gender ratios, illiteracy rates, maternal mortality rates and other health indicators in South Asian women.^128^ As opposed to the West, marriage does not seem to be a protective factor against suicide in Pakistan. This likely indicates the high level of marital stress married women face compared with single women.^129^ Ali et al identified the pursuit of higher education as an agent toward change for all genders in Pakistan. The authors also recognised the role of mass media in supporting women's empowerment.^125^

With the increasing availability of handheld devices and internet access in both urban and rural areas of Pakistan, the influence of social media on suicide behaviour cannot be disregarded. In a recent study, Cheng et al identified the role of social media and Facebook in depicting suicide and having an intended effect of similar choice of agent in other suicides.^130,131^ Others have focused on local newspapers and the impact of reporting suicide on the front pages.^132^

Religious beliefs can provide a series of effective coping strategies (e.g. prayer, rituals, religious services and social networks) that are considered as protective factors against suicide.^133^ A strong belief in God and that whatever happens is by Allah's will may create an atmosphere of acceptance rather than desperation in Muslims.^26^ Rezaeian argues that Islam attempts to address the underlying factors contributing to the suicidal state, such as promoting mental health by the remembrance of the creator (Zikr), decreasing poverty by the distribution of wealth through mandatory charity (Zakat), and forbidding alcohol and other intoxicants.^134^ Although religious beliefs and laws against suicide may be a deterrent, inadvertent negative consequences, such as a delay in help-seeking, fear of prosecution by the police and legal authorities, stigma and a lack of reliable statistics, can also occur.^30^ For religious families, suicide is viewed as a sin and a failure rather than an illness. It may dictate family reactions, treatment-seeking behaviours, explanations of disease and adherence to treatment.^39^ It is important to note that the clinicians’ own religious view of suicidal behaviour may lead to unconscious biases in delivering clinical care, and could lead to moral and ethical dilemmas when treating such patients.^30^

Recent literature has challenged the notion of outright faith-based protection. Pritchard et al explored ‘hidden’ or missed suicides in Islamic countries. They suggested that the official records seemed to be at odds with the study results purporting a higher number of suicides in Muslim-majority countries than previously reported.^39,135^ The authors identified the risk of the unrecognised or denied extent of suicidal behaviour, undermining the necessary steps to support the individual and prevent fatal outcomes. Similarly, Jordans et al found a higher reported suicide rate in South Asia, mainly driven by Bangladesh (a Muslim-majority country), India and Sri Lanka, compared with the global average.^136^

Our data did not report on individual risk factors for suicide. However, we include a brief overview to emphasise its importance in the study of suicide. Previous analyses showed poor impulse control, premorbid depression, a history of physical/sexual or emotional abuse, high risk-taking behaviour and low self-esteem as contributing toward self-harm and suicide.^41^ Cognitive factors such as low IQ and limited education; poor problem-solving or inadequate communication skills; lack of distress tolerance; and the timing of the attempt, such as after a similar attempt in the family or neighbourhood, may also have a significant effect on the choice of agent in self-poisoning.^19,137,138^ Copycat suicides or Werther's syndrome have long been identified as drivers of cluster suicides.^139–143^ Although we did not look for the timing of cluster suicides in our analysis, it should be explored in future studies.^113^ In short, the prevalence, characteristics and methods of suicidal behaviour vary widely between different communities, across other demographic groups and over time.^144^

Structural determinants of health account for some of the regional variations noted in our study.^145,146^ We argue that the easy and unrestricted availability of drugs/medicines could be one reason for these regional variations. Ali et al have raised concern about the lack of regulation for over-the-counter drugs in Pakistan, leading to misuse and overuse.^118^ The authors did not consider the risk of suicide overdose with uncontrolled access to medications, which we believe should be factored into future regulations. Pakistan's growing income inequality and increase in poverty are concerning.^146,147^ Li and Katikireddi emphasised the urban–rural inequalities as a driver of suicide trends.^148^ The efforts to decriminalise suicide in Pakistan gained momentum likely after India decriminalised suicide in 2015.^10,149,150^ Although Islam condemns those who commit suicide, no legal or societal punishment is mentioned for suicide survivors in the Quran.^122^ In February of 2018, the Pakistan Senate passed a bill for treatment of those who attempt suicide and survive, rather than punishment under Section 325 of the Pakistan Penal Code.^151^

With the alarming rise of suicide rates in Pakistan, we must emphasise urgent steps to halt and gradually reverse the suicide trends. It is imperative to initiate mental health literacy and psychoeducational campaigns in vulnerable communities, to identify high-risk individuals and the hazardous effects of agricultural chemicals.^152^ Furthermore, increasing the availability of resources for timely and prompt treatment of overdose may prevent dire consequences. The role of partnership with local leaders and utilisation of existing resources in such endeavours, such as governmental or non-governmental organisations, especially in rural areas, cannot be overemphasised.^153,154^ In a recent article, Eddleston and Gunnell focused on preventing suicide through regulating pesticides, especially in LMICs.^155^ Chowdhury et al reported the promising effects of a ban on class I pesticides in Bangladesh and a corresponding overall decrease in suicide rate in the region.^156^

Similarly, Sri Lanka and South Korea have achieved success through governmental regulations in the availability of pesticides and insecticides.^5,157,158^ With our collective effort, there is no reason that Pakistan could not achieve the same. After the next three decades, a strikingly different review focusing on suicide rate reduction success may be reported. As noted above, the Punjab Government has taken the first steps to ban potentially harmful agents.^115^ The Federal Government of Pakistan must follow suit in steering the campaign against suicide in the right direction. In a recent paper, Zia emphasised the need for clear warning labels, phrases in local languages and symbols on pesticides and other hazardous chemicals. The author suggested that the advertisement must include safety warnings as for cigarettes, and a strict following of Food and Agriculture Organization of the United Nations guidelines should be implemented.^159^ The need for systemic media campaigns for awareness and safe pesticide is necessary. We believe that despite the recent step of passing the decriminalisation of suicide bill in the Senate, it will take a concerted effort to decrease stigma against suicide survivors.

### Management of individual agents

A summary of the management of agents is as follows (see Table 5):

#### Organophosphates

Organophosphate compounds are a diverse group of chemicals used in domestic, industrial and agricultural settings. Examples include insecticides and pesticides (malathion, parathion, etc.), herbicides (glyphosate, atrazine, etc.) and nerve gases (sarin, tabun, VX).^160^ Organophosphate poisoning is one of the most common methods used for suicide, and is a leading cause of death in young people in Pakistan, China, India, Sri Lanka and other Asian countries.^161–163^ It is recognised as the principal mode of poisoning in southern Punjab, and accounts for 47–60% of instances reported in Sindh.^164–166^ Data from other parts of the country suggest organophosphates as a cause of poisoning in 20 to 40% of cases.^50,54,165^

Inhalation, ingestion or skin contact can lead to organophosphate poisoning. The organophosphate molecule binds and inactivates acetylcholinesterase enzyme in red blood cells. This leads to an overabundance of acetylcholine within both nicotinic and muscarinic synapses and the neuromuscular junctions.^166^ The nicotinic effects are rapid in onset and may include twitching of fine muscles, fasciculations and hyperreflexia, which may progressively lead to flaccid paralysis. Muscarinic receptors are located in both the sympathetic and parasympathetic nervous systems, and are usually slower in onset because of their action via G-protein-coupled receptors. Symptoms such as bronchorrhea, bronchoconstriction, excessive sweating, constricted pupils, abdominal cramps, involuntary defaecation and urination, tachycardia, QT prolongation, headaches, dizziness, drowsiness, confusion, anxiety, slurred speech, ataxia, psychosis, convulsions, coma, hypotension and respiratory depression can occur.^167–169^

The diagnosis of organophosphate poisoning is clinical and based on the presenting history, collateral information from the attendants and the clinical signs. Confirmation of organophosphate poisoning can be obtained by measuring plasma butyrylcholinesterase activity or acetylcholinesterase in whole blood; however, such assays are not readily available to inform clinical decision-making.^170^ The first step is to decontaminate the patient and prevent further absorption via the eyes, skin or lungs. Personal protective equipment must be used to avoid exposure. The standard treatment of organophosphate poisoning is the reversal of muscarinic manifestations using atropine, followed by enzyme reactivation by pralidoxime. Frequent atropine doses or continuous infusion are used to clear excessive respiratory secretions and to treat bradycardia.^171^ Atropine should be continued for 1–3 days after successful atropinisation. Pralidoxime facilitates the recovery of neuromuscular transmission at the nicotinic synapses. It significantly reduces atropine consumption in organophosphate poisoning, and signs of atropinisation may occur earlier with its use than without its administration.^172^

In our analysis, mortality ranged from 10 to 27%. It was dependent on the amount of substance ingested, the time to reach an emergency department or time to initiation of treatment, and the use of a ventilator for assisted breathing.^173^ Other predictors of mortality include age >40 years, bradycardia, low pH, high glucose, high lactate dehydrogenase and low Glasgow Coma Scale score.^44,174^

#### Aluminium phosphide

Aluminium phosphide is a highly toxic, solid fumigant insecticide and rodenticide used for grain conservation.^175^ It is referred to as wheat pills in Pakistan, and is also known as rice pills or rice tablets in other countries.^176,177^ It is not regulated by the government and is available for over-the-counter purchase without any restriction, making it an ideal agent for self-poisoning in the wheat-growing areas of northern and central Punjab.^178^ Studies have reported its use as an agent of suicide by ingestion from Rawalpindi,^178^ Kharian,^75^ Lahore,^179^ Sahiwal^78^ and Peshawar in KPK.^54^ In these areas, domestic conflicts or petty quarrels are a frequent cause of overdose, resulting in fatal outcomes.^78^ The lack of an antidote makes it a prevalent and particularly lethal suicide agent.^175^

When exposed to moisture in the stomach after ingestion, phosphine gas is produced. This toxic gas inhibits cytochrome c oxidase and other vital cellular enzymes, disrupting several metabolic pathways and destabilising cell membranes. Disruption of mitochondrial function produces reactive hydroxyl radicals, leading to cellular hypoxia, free-radical-mediated injury and eventual cell death.^176,180^ The presenting symptoms of aluminium phosphide poisoning may include epigastric pain, vomiting, diarrhoea, dizziness and dyspnoea.^178^ Multiorgan failure involving the heart, kidneys, lungs and liver later ensues, with metabolic acidosis, hepatic necrosis, renal failure, cardiac arrhythmia, congestive heart failure and hypotensive shock.^180,181^

A silver nitrate test can be performed to confirm the diagnosis. Paper impregnated with silver nitrate turns black after exposure to the patient's breath or gastric contents, as a result of the reaction between phosphides and silver nitrate. The sensitivity of the test strip is 50% with a breath test and 100% with gastric contents.^182^

The treatment is supportive because of the absence of an antidote. Gastric lavage with potassium permanganate and mineral or coconut oil has been shown to reduce morbidity.^183^ Besides symptomatic treatment, renal replacement therapy in the early stage is also recommended.^184^

Aluminium phosphide is termed ‘agent of sure death’,^185^ and the mortality rate ranged from 33 to 87% in our data.^186^ The lethal dose for an adult is 150–500 mg. The presence of vomiting, exposure of tablets before ingestion and early availability of supportive care can help decrease mortality.

#### Paraphenylenediamine

Paraphenylenediamine is an ingredient of a compound commonly known as *kala pathar* or ‘Black Stone’ in Urdu. It is used as a chemical ingredient in temporary tattoo ink, fabrics, dark makeup, photocopying inks, printing, rubber products and gasoline. In the Indian subcontinent and North Africa, it is an ingredient of black henna for hair dye and tattoo ink.^84–86^ Paraphenylenediamine is metabolised into benzoquinone diamine by cytochrome P450 peroxidase, and further oxidation results in the formation of Brandowaski's base. Both of these by-products are responsible for their toxicity.^187,188^ Paraphenylenediamine ingestion is another conventional means to commit suicide in southern Punjab.^189^

The most common clinical presentations after paraphenylenediamine intoxication include cervicofacial oedema, rhabdomyolysis causing myoglobinuria, cola-coloured urine, oliguria and acute tubular necrosis leading to renal failure.^190^ A study of 150 cases of paraphenylenediamine poisoning from Sudan revealed angioneurotic oedema and conjunctival discolouration in 100% of cases, and acute kidney injury requiring haemodialysis in 60% of cases.^191^

There is no antidote available for paraphenylenediamine poisoning. As the chemical is nondialysable, the mainstay of management remains supportive.^192^ The patient must be observed in the intensive care unit. Management includes early tracheostomy for cervicofacial oedema and intravenous fluids, with aggressive diuresis and urine alkalisation for renal failure.^193–195^ Rhabdomyolysis may lead to acute tubular necrosis, requiring haemodialysis.

The outcome of paraphenylenediamine ingestion depends on the dose taken. The lethal dose of paraphenylenediamine is unknown, and estimates vary from 7 to 10 g.^196,197^ A large quantity (>7 g) might cause death within the first 6–24 h from angioneurotic oedema or cardiotoxicity.^198^ The mortality ranges from 21 to 47%.

#### Others

This group included over-the-counter agents, prescription medicine, agents of abuse and household chemicals. This type of poisoning was more common in young patients (15–35 years) from urban backgrounds.^50,76,77,91,199^ Males overdosed at a higher rate than females.^91,199^ Benzodiazepines were the most common agent used for overdose;^91,116,197^ however, other agents used were NSAIDs, analgesics, sedatives, tricyclics, anti-emetics, antiallergics, anti-epileptics, oral hypoglycaemics, warfarin, digoxin, methamphetamine and cocaine.^50,91,197^ Corrosives, kerosene oil, rubbing alcohol, copper sulphate, bleach, rat poison pills and home insecticide sprays were also used.^63,76,77,91,199^ Most patients taking an overdose had an intention to commit suicide; however, other reasons for overdose were to gain attention, express distress or get revenge.^50,200^ The researchers interviewed a total of 80 individuals admitted after suicide overdose, to determine their intention to die, and noted that the patients with such an intention chose organophosphates because of its known high lethality.

The most common presentation was drowsiness owing to central nervous system depression; others presented with central nervous system stimulation or a mixed picture.^91^ Heart rate, blood pressure, body temperature, respiratory rate, skin clamminess, pupillary reaction and neuromuscular abnormalities provided clues to the correct diagnosis.

Treatment includes decontamination and gastric lavage with activated charcoal. The use of the benzodiazepine antidote flumazenil remains controversial as it could precipitate withdrawal seizures in individuals who have developed tolerance from chronic use.^201^ Flumazenil use in paediatric benzodiazepine overdose may be used as young children are unlikely to be tolerant to benzodiazepines.^202^ Haemodialysis, haemofiltration and exchange transfusion could facilitate removing the agents or chemicals from circulation. Supportive care is indicated for strict airway monitoring, gastrointestinal protection and the treatment of hypo- or hypertension.

Mortality varied from 2.5 to 25%, depending on the place of study. General medical wards reported lower death rates than intensive care units, likely related to the severity of the patient's condition.^50,54^ Mortality was also dependant on the level of care available in the centre where the patient was under treatment.

There are several limitations to this analysis. We considered papers in the English language, from only two electronic databases, and excluded single case reports in this retrospective analysis. Significant variations in the reported information in descriptive studies make it difficult to analyse or present the data in a meta-analysis. Limited data were available from Balochistan and Gilgit–Baltistan, and studies from other provinces also represented only larger cities. Risk factors were not available for extensive analysis. More comprehensive studies are required to explore how individual differences influence regional trends of suicide and other means of suicide that were not addressed in our review.

### Summary and future directions

Our study found that there are striking regional and urban versus rural differences in the choice of agents used for suicide. As the suicide rate in Pakistan is rapidly increasing, we must take several steps to reverse the trend of the past three decades. We should launch customised mental health literacy and public health awareness campaigns across the country, to address the stigma against suicide and mental health. The success and acceptance of such endeavours will depend on partnership with local authorities, tribal or clan leaders, religious leaders and influential community figures. Print (newspapers, magazines, etc.), electronic (network television, radio, etc.) and social media (Facebook, Twitter, Instagram, YouTube, etc.) may enhance the dissemination of the message. Efforts are needed to enforce the existing national pesticide policy. There is a need to have regulations to restrict over-the-counter sales of potentially dangerous medications, such as benzodiazepines, opiates and opioid derivatives. Finally, our hospitals need consistent medical supplies and specialised equipment, along with training of medical staff, to manage victims adequately. These interventions are necessary to reduce morbidity and mortality related to suicide poisoning in this time of crisis.

## Data availability

The authors confirm that the data supporting the findings of this study are available within the article.
